# Stabilization of cytokine mRNAs in iNKT cells requires the serine-threonine kinase IRE1alpha

**DOI:** 10.1038/s41467-018-07758-x

**Published:** 2018-12-17

**Authors:** Srinath Govindarajan, Djoere Gaublomme, Renée Van der Cruyssen, Eveline Verheugen, Sofie Van Gassen, Yvan Saeys, Simon Tavernier, Takao Iwawaki, Yehudi Bloch, Savvas. N. Savvides, Bart N. Lambrecht, Sophie Janssens, Dirk Elewaut, Michael B. Drennan

**Affiliations:** 1 Unit for Molecular Immunology and Inflammation, VIB Center for Inflammation Research, Technologiepark 927, 9052 Zwijnaarde (Ghent), Belgium; 2 Department of Rheumatology, Ghent University, Ghent University Hospital, Ghent, 9000 Belgium; 3 Department of Applied Mathematics, Computer Science and Statistics, Ghent University, Ghent, 9000 Belgium; 4 Data Mining and Modeling for Biomedicine, VIB Center for Inflammation Research, Technologiepark 927, 9052 Zwijnaarde (Ghent), Belgium; 5 Laboratory of Immunoregulation and Mucosal Immunology, VIB Center for Inflammation Research, Technologiepark 927, 9052 Zwijnaarde (Ghent), Belgium; 6 Department of Respiratory Medicine, Ghent University, Ghent University Hospital, 9000 Ghent, Belgium; 7 Division of Cell Medicine, Department of Life Science, Medical Research Institute, Kanazawa Medical University, Kanazawa, 920-0856 Japan; 8 Unit for Structural Biology, Department of Biochemistry and Microbiology, Ghent University, Technologiepark 927, 9052 Zwijnaarde (Ghent), Belgium; 9 Unit for Structural Biology, VIB Center for Inflammation Research, Technologiepark 927, 9052 Zwijnaarde, (Ghent), Belgium; 10 Department of Pulmonary Medicine, Ghent University, ErasmusMC, Rotterdam, 2040 Netherlands; 11 Laboratory of ER Stress and Inflammation, VIB Center for Inflammation Research, Technologiepark 927, 9052 Zwijnaarde (Ghent), Belgium; 12 Department of Internal Medicine and Pediatrics, Ghent University, Ghent, 9000 Belgium

## Abstract

Activated invariant natural killer T (iNKT) cells rapidly produce large amounts of cytokines, but how cytokine mRNAs are induced, stabilized and mobilized following iNKT activation is still unclear. Here we show that an endoplasmic reticulum stress sensor, inositol-requiring enzyme 1α (IRE1α), links key cellular processes required for iNKT cell effector functions in specific iNKT subsets, in which TCR-dependent activation of IRE1α is associated with downstream activation of p38 MAPK and the stabilization of preformed cytokine mRNAs. Importantly, genetic deletion of IRE1α in iNKT cells reduces cytokine production and protects mice from oxazolone colitis. We therefore propose that an IRE1α-dependent signaling cascade couples constitutive cytokine mRNA expression to the rapid induction of cytokine secretion and effector functions in activated iNKT cells.

## Abstract (web summary)

Invariant natural killer T (iNKT) cells rapidly enhance cytokine secretion and effector function following activation, but the underlying mechanism is still unclear. Here the authors show that an endoplasmic reticulum stress sensor, inositol-requiring enzyme 1α, activates the p38 kinase to stabilize cytokine mRNA for enhanced iNKT functions.

## Introduction

Invariant natural killer T (iNKT) cells are characterized as an innate T-cell subset that recognizes glycolipid antigens presented by CD1d, an MHC class I-related molecule^1^. A hallmark feature of iNKT cells is their ability to rapidly produce and secrete immunomodulatory cytokines following T-cell receptor (TCR) ligation, implicating them in a range of inflammatory, allergic, and autoimmune diseases^1^. Although this functional aspect of iNKT cell biology is not fully understood, it has been suggested that the presence of preformed cytokine mRNAs as well as histone acetylation of distinct cytokine loci facilitate rapid iNKT cell cytokine production^2,3^. However, beyond such studies, it has proved difficult to investigate the potential regulatory mechanisms involved in iNKT cell cytokine production as many of these signaling pathways also control iNKT cell development, maturation, and survival^1,4^. We therefore sought to investigate whether iNKT cells utilize components of the unfolded protein response (UPR) to accommodate the rapid increase in cytokine production following activation as has been observed for the production of antibodies during plasma cell differentiation^5,6^.

UPR is an intracellular signal transduction pathway conserved from yeast to mammals that senses perturbations in protein folding, protein synthesis and/or calcium homeostasis within the endoplasmic reticulum (ER). In mammals, the UPR consists of the three proximal ER stress sensors; inositol-requiring enzyme 1α (IRE1α), ER-resident protein kinase R-like endoplasmic reticulum kinase (PERK), and activating transcription factor 6 (ATF6) that collectively function to promote ER homeostasis by increasing protein folding capacity and protein biosynthesis within the ER during stress^7^. Prolonged or severe ER stress that cannot be resolved by induction of the UPR is widely considered to trigger apoptosis and inflammation and is involved in the development of a number of human diseases characterized by a metabolic or inflammatory pathology^8^.

IRE1α is a type I ER-resident transmembrane protein that comprises an ER luminal and cytosolic domain with both serine-threonine kinase and endoribonuclease activity^9^. During ER stress, oligomerization of the luminal domain of IRE1α results in autophosphorylation of the cytosolic domain and activation of a sequence-specific endoribonuclease (RNase) which recognizes and cleaves an intron from pre-mRNA encoding the bZIP transcription factor XBP1^10^. Translocation of cleaved or spliced XBP1 (XBP1s) to the nucleus is associated with the upregulation of ER chaperone proteins and enzymes which function to increase protein folding capacity and quality control within the ER^11,12^. The RNase domain of IRE1α also targets and degrades distinct mRNAs containing a consensus sequence in a process termed regulated IRE1α-dependent decay (RIDD)^13^, further reducing protein translocational load during ER stress. In addition to these functions, autophosphorylation of IRE1α during UPR is also associated with downstream c-Jun kinase (JNK) phosphorylation^14^, which is proposed to promote apoptosis in cells unable to resolve ER stress^15^. ER stress however also activates additional ER stress sensors including the protein kinase PERK and the transcription factor ATF6^16,17^. Here, the substrates for the protein kinase activity of PERK have been identified, namely the eukaryotic translation initiation factor 2α (eIF2α). EIF2α has been shown to counteract the formation of reactive oxygen species and to inhibit cap-dependent mRNA translation^18^. ER stress-mediated proteolysis of the ER luminal domain of ATF6 results in the liberation of a bZIP transcription factor that induces genes involved in ER chaperone function and ER-associated protein degradation (ERAD)^17,19^. Collectively, UPR therefore promotes ER homeostasis and cell survival by regulating an adaptive response at both the transcriptional as well as translational level. Irremediable ER stress is however associated with inflammatory signaling and the initiation of apoptosis^15^.

Although it is widely acknowledged that UPR regulates cellular survival during ER stress, several studies have shown that UPR is also required by B lymphocytes during plasma cell differentiation^5,20^. Here, XBP1s is required for the increased synthesis and secretion of Ig chains by plasma cells^5,6^, suggesting that IRE1α is directly activated during B-cell differentiation. Similarly, T-cell differentiation following TCR ligation is associated with the activation of IRE1α^21^, whereas PERK has been shown to control T-cell effector functions by regulating the translation of distinct cytokine mRNAs^22^.

In this study, we demonstrate that defined iNKT sublineages constitutively express components of the UPR, and require IRE1α to stabilize cytokine mRNAs following activation both in vitro and in vivo. We propose that these findings represent a novel mechanism whereby IRE1α functions as a central regulator of cytokine production for specific iNKT subsets in vivo.

## Results

### IRE1α RNase domain is active within NKT1 and 17 sublineages

**Fig. 1:**
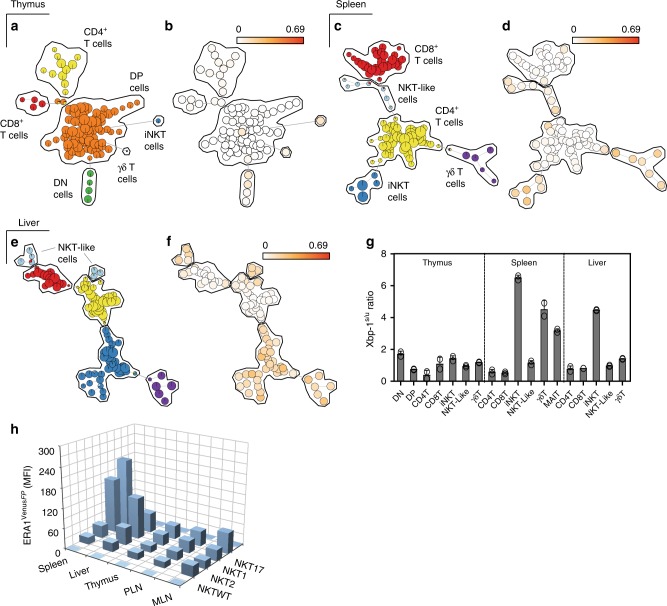
Constitutive IRE1α RNase activity in iNKT cells of naïve ERA1- mice. **a**−**f** Minimal spanning tree visualization of T-cell subsets by FlowSOM analysis. Manual gating results for **a** thymus, **b** spleen and **c** liver indicate which nodes correspond to the indicated T-cell subsets. Node size corresponds to the percentage of cells represented by each node. **b**, **d**, **f** *Venus*^FP^ expression within ERA1^FP/WT^ and control ERA1^WT/WT^ mice for thymus, spleen, and liver, respectively (*n* = 3). Data represent three independent experiments. **g** Xbp-1s/u mRNA ratio expressed by T-cell subsets isolated from thymus, spleen, and liver of C57Bl/6 mice (*n* = 5). Bar chart represents two independent experiments pooled. **h** *Venus*^FP^ MFI within NKT1, 2 and 17 cells isolated from thymus, spleen, PLN, MLN, and liver of ERA1^FP/WT^ mice (*n* = 3). iNKT^WT^ represents *Venus*^FP^ MFI within control iNKT cells isolated from WT mice. Bar chart represents one of three independent experiments. T lymphocyte subsets in (**a**−**g**) are defined in Materials and Methods. Error bars show the mean ± s.e.m

To assess steady-state UPR activity in T cells by flow cytometry, we analyzed expression of a variant of green fluorescent protein (FP) in T cells isolated from the thymus, spleen and liver of naïve ERAI-transgenic (ERAI^FP/WT^) mice^23^. We utilized ERAI^FP/WT^ mice as a UPR-specific reporter as they express a partial sequence of human XBP-1 that includes the sites at which IRE-1α splices XBP-1, fused to *Venus* fluorescent protein (*Venus*^FP^), which allows reporting of the IRE-1α arm of the UPR^23^. Subsequent flow cytometric analysis of the T-cell compartment using a Self-Organizing Map (FlowSOM)^24^ visually defined specific T-cell lineages as a minimal spanning (MS) tree in which clusters of similar cells were represented as nodes within each T-cell subset (Fig. 1a, c, e). In this manner, the MS trees clearly identified distinct T-cell subsets within each organ as either double-negative (DN, green) or double-positive (DP, orange) thymocytes, CD4^+^ T cells (yellow), CD8^+^ T cells (red), CD1d tetramer-negative NKT-like cells^25^ (light blue), iNKT cells (dark blue) and γδ T cells (purple), respectively (Fig. 1a, c, e). *Venus*^FP^ expression within each node was subsequently determined by computing the difference between *Venus*^FP^ expression within T cells isolated from ERAI^FP/WT^ mice and background auto-fluorescence in T cells isolated from littermate control (ERAI^WT/WT^) mice. As is shown in Fig. 1b, d, f, the majority of *Venus*^FP^ expression was found to be restricted to iNKT cells present in the spleen and liver; however, reporter expression was also detected within peripheral iNKT and γδ T-cell lineages (Fig. 1c, e). Data visualization of *Venus*^FP^ expression using the FlowSOM MS tree was therefore independently examined by analyzing the expression of spliced and unspliced XBP1 mRNA within FACS-sorted T-cell subsets isolated from thymus, spleen, and liver of wild-type mice. Here, qPCR analysis verified that splenic and hepatic iNKT cells expressed the highest level of IRE1α RNase activity when compared to γδ and T-cell lineages (Fig. 1g). A subsequent analysis of *Venus*^FP^ expression within the NKT1, 2 and 17 sublineages as defined by the cell-surface markers CD27 and CD122^26,27^ revealed selective expression of *Venus*^FP^ within the NKT1 and NKT17 sublineages, respectively (Fig. 1h). Notably, only NKT17 cells present in the spleen expressed *Venus*^FP^ whereas NKT2 cells isolated from the thymus, spleen, liver, popliteal lymph node (PLN) and mediastinal lymph node (MLN) did not (Fig. 1h). These results therefore show that a constitutively active IRE1α RNase is present within the peripheral iNKT cell compartment and that splenic and hepatic NKT1 and NKT17 sublineages express the highest levels of XBP1s.

### TCR ligation induces UPR in NKT1 and 17 sublineages

**Fig. 2:**
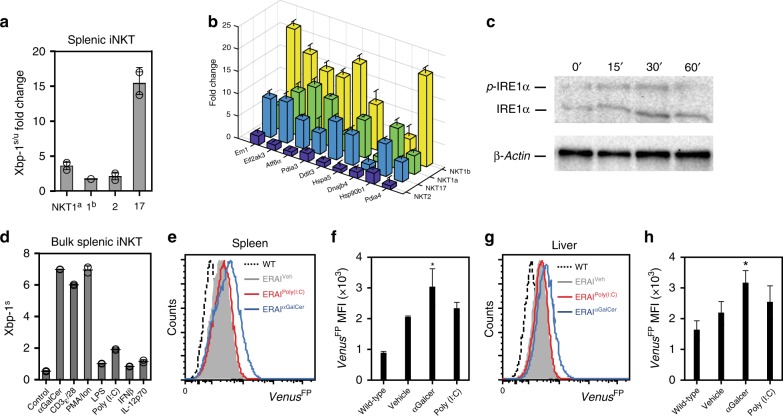
TCR-dependent stimuli regulate activation of UPR in NKT sublineages. Expanded splenic NKT cell sublineages were restimulated with anti-CD3/CD28 (3 μg.ml^−1^/5 μg.ml^−1^) for 3 h and fold induction of Xbp-1s/u (**a**) and ER chaperone mRNAs (**b**) in stimulated versus resting cells was assessed by qPCR (*n* = 3). Bar chart represents two independent experiments pooled. **c** Analysis of phosphorylated IRE1α and total IRE1α in bulk expanded splenic iNKT cells restimulated with α-CD3ε/CD28 for the indicated times via phos-tag immunoblot assay. β-Actin represents the loading control. Results represent one of two independent experiments. **d** qPCR analysis of XBP1s in expanded splenic iNKT cells stimulated with α-GalCer (100 ng/ml), anti-CD3/CD28 (3 μg.ml^−1^/5 μg.ml^−1^), PMA/Ionomycin (1 μΜ/200 ng.ml^−1^), LPS (20 ng/ml), Poly(I:C) (20 ng/ml), IFNβ (100 U/ml), IL-12p70 (20 ng/ml) or medium (*n* = 3). Bar chart represents two independent experiments pooled. **e**−**h** Control and ERA1^FP/WT^ mice were injected with either vehicle, α-GalCer or Poly(I:C) and iNKT cells were analyzed in spleen and liver 2 h later. Histograms and bar graphs represent *Venus*^FP^ expression within iNKT cells isolated from the spleen and liver of WT (dashed line, *n* = 2), vehicle-injected ERA1^FP/WT^ (gray, *n* = 3), α-GalCer-injected ERA1^FP/WT^ (blue line, *n* = 3) or Poly(I:C)-injected ERA1^FP/WT^ mice (red line, *n* = 3) at 2 h postinjection. Histogram and bar chart represents one of three independent experiments. Error bars show the mean ± s.e.m. *p < 0.05 determined by Mann–Whitney *U* test

iNKT cells have been shown to be activated by signals emanating from their TCR^1^ as well as by cytokines such as IL-12 and type 1 IFN^28,29^. We initially examined the role of TCR-dependent signaling in regulating IRE1α RNase activity within iNKTs by stimulating splenic iNKT sublineages in vitro using anti-CD3/CD28. For these experiments, splenic NKT1^a^, NKT1^b^, NKT2, and NKT17 sublineages were FACS sorted based on CD27 and CD122 cell-surface expression and expanded in vitro as has been previously reported^26,27^. Subsequent in vitro restimulation of iNKT sublineages revealed that TCR-dependent stimulation induced pronounced splicing of *Xbp1* mRNA (Fig. 2a), as well as several UPR-associated mRNAs (Fig. 2b) within the NKT17 and NKT1, but not NKT2 sublineage cells. Importantly, we show that TCR-induced splicing of *Xbp1* mRNA within restimulated iNKT cells (Fig. 2a) is associated with increased autophosphorylation of IRE1α (Fig. 2c), suggesting that TCR-mediated signaling within iNKT cells induces activation of the kinase domain of IRE1α. A subsequent analysis of the ability of additional stimuli to induce *Xbp1* mRNA splicing within in vitro expanded iNKT cells revealed that neither cytokines such as IL-12p70 and IFNβ, nor Toll-like receptor (TLR) agonists such as lipopolysaccharide (LPS) or Poly (I:C) induced significant IRE1α RNase activity above that observed for medium controls (Fig. 2d). These results were confirmed in vivo using ERAI^FP/WT^ mice (Fig. 2e−h). Here, intraperitoneal (i.p.) administration of α-galactosylceramide (α-GalCer), a TCR ligand, significantly increased *Venus*^FP^ expression within splenic and hepatic iNKT cells when compared to vehicle controls (Fig. 2e−h). In contrast, induction of type 1 IFN following administration of Poly (I:C) failed to modulate expression of *Venus*^FP^ within iNKTs isolated from ERAI^FP/WT^ mice (Fig. 2e−h). Thus, TCR-dependent stimuli induce UPR and IRE1α RNase activity within the NKT1 and NKT17 sublineages, both in vitro and in vivo.

### iNKT sublineage development is IRE1α-independent

IRE1α has previously been shown to be required in pre-B cells during B-cell receptor formation as well as for the secretion of immunoglobulins (Igs) during plasma cell differentiation^5,30^. In contrast, development and activation of the conventional T-cell compartment is unaltered in the absence of IRE1α^31^, suggesting that IRE1α regulates lymphocyte development and function in a lineage-specific manner. We therefore sought to determine whether iNKT cell development required IRE1α as iNKT cells express a constitutively active IRE1α RNase (Fig. 1), and upregulate spliced XBP1 mRNA following TCR stimulation (Fig. 2). To examine the requirement for IRE1α during iNKT cell development, we generated CD4 T-cell-specific IRE1α-deficient mice (CD4^cre^;IRE1α^Δ/Δ^) by intercrossing CD4-cre transgenic mice with mice carrying a LoxP flanked IRE1α allele. A subsequent analysis of the frequency and absolute cell counts of iNKT cells present in the thymus, spleen, and liver of CD4^cre^;IRE1α^Δ/Δ^ mice revealed no significant differences when compared to littermate IRE1α^F/F^ controls (Supplementary Fig. 1A, B), and the development and maturation of iNKT cell thymocytes from stage 1 (CD44^lo^NK1.1^−^) to stage 3 (CD44^hi^NK1.1^hi^) iNKT cells occurred normally in the absence of IRE1α (Supplementary Fig. 1C, D). Similarly, analysis of iNKT sublineage development using the transcription factors promyelocytic leukemia zinc finger (PLZF) and RORγt revealed no significant differences in the frequency or absolute numbers of NKT1, NKT2 or NKT17 sublineages present in thymus, spleen, and liver of CD4^cre^;IRE1α^Δ/Δ^ mice relative to controls (Supplementary Fig. 1E, F). To directly evaluate the proliferative capacity of iNKT cells in the absence of IRE1α, DNA labeling studies with BrdU were performed after administration of mice intraperitoneally with α-GalCer. Analysis of iNKT cells expansion after 5 days postinjection with α-GalCer revealed no significant differences between CD4^cre^;IRE1α^Δ/Δ^ mice when compared to littermate controls (Supplementary Fig. 1G). Thus, IRE1α serves a limited role in the selection, maturation, and lineage specification of the iNKT cell compartment in steady-state animals.

### iNKT cell motility dynamics are IRE1α-independent

To further analyze potential differences between wild-type and IRE1α-deficient iNKT cells, intravital motility dynamics of hepatic iNKT cells were investigated using multiphoton microscopy. For these experiments CD4^cre^;IRE1α^Δ/Δ^ mice were backcrossed onto a CXCR6^gfp/−^ background as this mouse line has previously been shown to be a reporter specific for hepatic iNKT cells^1^. CXCR6^gfp/−^IRE1α^F/F^ (IRE1α^WT^) and CXCR6^gfp/−^CD4^cre^;IRE1α^Δ/Δ^ (IRE1α^KO^) mice were subsequently imaged for 2 h at steady-state until they received an i.v. injection with α-GalCer, after which they were imaged for two more hours to capture the TCR-mediated cell arrest that follows iNKT cell activation^32^. The overall appearance and behavior of IRE1α^WT^ and IRE1α^KO^ mice were very similar (Supplemental movies 1 and 2), apart from the sporadic presence of clusters of GFP^+^ cells in the KO mice. We quantified the motility dynamics and showed that there was no significant difference in the average speed (Supplementary Fig. 2A), speed distribution (Supplementary Fig. 2B), cell track distribution (Supplementary Fig. 2C-F), displacement over time (Supplementary Fig. 2G), and confinement index (Supplementary Fig. 2H) of GFP^+^ cells in IRE1α^WT^ and IRE1α^KO^ mice, respectively. Plotting the squared displacement over time allows to differentiate between different types of migration, with a linear curve representing random movement, whereas for example an upwards curve reveals directed movement. The confinement index is a measure of the straightness of the cell tracks, with a low value indicating confined movement, possibly caused by interaction with neighboring cells. As expected, iNKT cells show a more confined movement following injection with α-GalCer. Overall, IRE1α does not appear to influence iNKT cell dynamics.

### IRE1α specifically regulates NKT1 and 17 cytokine production

**Fig. 3:**
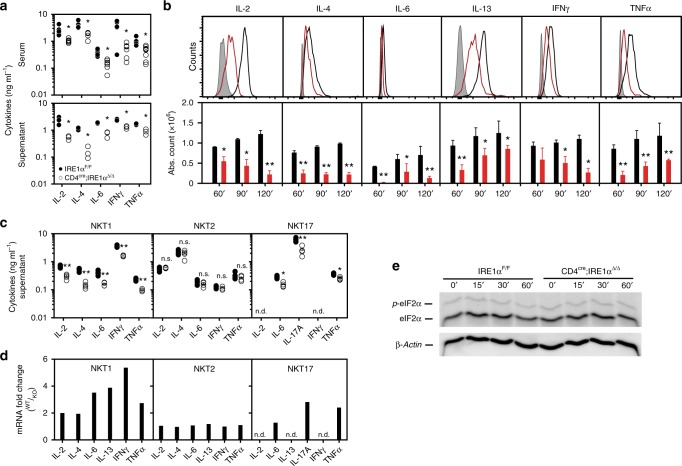
Impaired cytokine production by IRE1α-deficient iNKT cells. **a** Cytokines measured in serum from control (*n* = 11) and CD4^cre^;IRE1α^Δ/Δ^ (*n* = 12) at 4 h post-α-GalCer injection. Dot blots are representative of two independent experiments pooled. (Lower panel) Expanded iNKT splenocytes from control (*n* = 4) and CD4^cre^;IRE1α^Δ/Δ^ (*n* = 4) mice were restimulated with anti-CD3/CD28 for 72 h and cytokines were measured in supernatant by ELISA. **b** FACS histograms (upper panel) and absolute cell counts (lower panel) of cytokine-positive splenic iNKT cells isolated from control (black line, *n* = 4) and CD4^cre^;IRE1α^Δ/Δ^ (red line, *n* = 4) mice following injection of vehicle (isotype, gray) or α-GalCer at 60, 90, and 120 min postinjection, respectively. Histograms and bar charts are representative of two independent experiments. Supernatant cytokines (**c**) and cytokine mRNAs (**d**) from splenic NKT sublineages isolated from control (*n* = 4) and CD4^cre^;IRE1α^Δ/Δ^ (*n* = 4) mice following restimulation with anti-CD3/CD28 for 3 h. qPCR data are represented as fold mRNA change in control versus knock-out (KO) cells. Dot blot and bar chart represents two independent experiments pooled. Phos-tag immunoblot analysis of eIF2α and phosphorylated-eIF2α (**e**) in bulk expanded splenic iNKT cells isolated from control (*n* = 4) and CD4^cre^;IRE1α^Δ/Δ^ (*n* = 4) mice following restimulation with anti-CD3/CD28 at indicated time points. β-Actin represents the loading control. Results are representative of two independent experiments. Error bars show the mean ± s.e.m. *p < 0.05, **p < 0.01 determined by Mann–Whitney *U* test and unpaired *t* test; n.d. not detected; n.s. not significant

To investigate whether UPR-mediated signaling regulates the rapid secretion of effector cytokines by iNKT cells, we injected control and CD4^cre^;IRE1α^Δ/Δ^ mice intraperitoneally with α-GalCer. Analysis of serum cytokines at 4 h postinjection revealed significantly reduced levels of IL-2, IL-4, IL-6, IFNγ, and TNFα in CD4^cre^;IRE1α^Δ/Δ^ mice compared to littermate controls (Fig. 3a, upper panel). Similarly, significantly fewer cytokine-positive iNKT cells were detected in the spleens of CD4^cre^;IRE1α^Δ/Δ^ mice following injection of α-GalCer relative to controls (Fig. 3b), and bulk splenic IRE1α-deficient iNKT cells restimulated in vitro secreted significantly less cytokines than did control iNKT cells (Fig. 3a, lower panel). Subsequent FACS sorting and expansion of splenic iNKT sublineages from control and CD4^cre^;IRE1α^Δ/Δ^ mice revealed that NKT1 and NKT17 cells required IRE1α for the production of cytokines following TCR restimulation (Fig. 3c), as well as for the expression of cytokine mRNAs (Fig. 3d). In contrast, neither NKT2 cells nor polarized conventional CD4^+^ T cells showed a requirement for IRE1α in the expression and translation of cytokine mRNAs (Fig. 3c, d, and Supplementary Fig. 3A-C), suggesting that IRE1α regulates cytokine mRNA expression within the NKT1 and NKT17 sublineages.

Genetic deletion of IRE1α has previously been shown to result in activation of the PERK signaling pathway^33^ which is noteworthy as PERK promotes phosphorylation of eIF2α thereby preventing cap-dependent translation of many cellular mRNAs. Reduced cytokine levels in IRE1α-deficient iNKT cells may therefore have been a consequence of enhanced PERK-eIF2α activity. To control for this, we sorted and expanded bulk splenic iNKT cells from control and CD4^cre^;IRE1α^Δ/Δ^ mice and restimulated them in vitro with anti-CD3/CD28 (Fig. 3e). Importantly, both control and IRE1α-deficient iNKT cells expressed identical intracellular levels of eIF2α and phosphorylated eIF2α (Fig. 3e) following TCR-mediated restimulation, confirming that reduced cytokine levels in IRE1α-deficient iNKT cells is not a consequence of a general inhibition in mRNA translation.

### Cytokine mRNA stability within NKT1 and 17 requires IRE1α

**Fig. 4:**
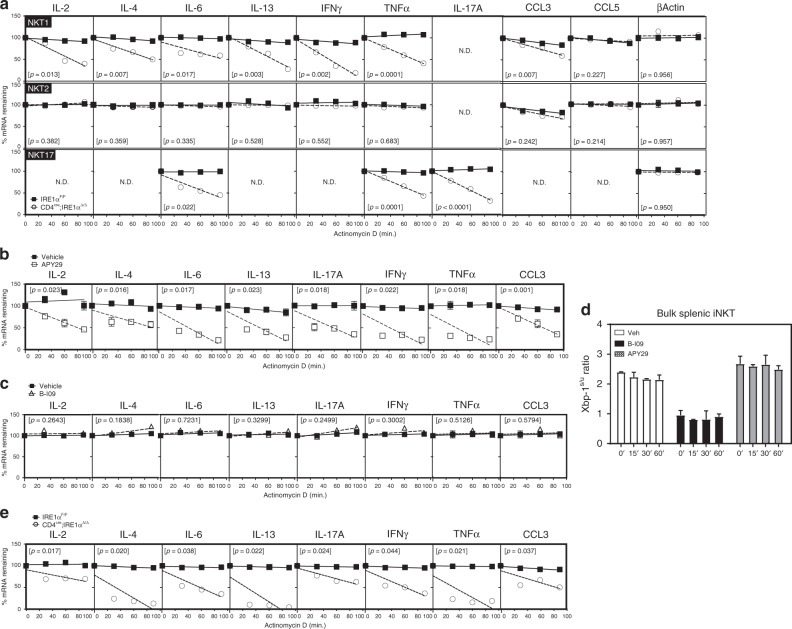
Reduced cytokine mRNA stability in IRE1α-deficient iNKT cells. **a** Splenic NKT1, 2 and 17 cells were isolated from control (*n* = 4) and CD4^cre^;IRE1α^Δ/Δ^ (*n* = 4) mice, expanded and restimulated with anti-CD3/CD28 for 3 h prior to addition of actinomycin D. mRNA decay rates were determined by linear regression analysis. Data represent three independent experiments pooled. **b**−**d** Bulk splenic wild-type iNKT cells were expanded in vitro, restimulated with anti-CD3/CD28 for 3 h in the presence of APY29 and B-109, and then treated with actinomycin D. mRNA decay rates were determined as above. Data represent two independent experiments pooled. **e** Purified bulk splenic iNKT cells from control (*n* = 6) and CD4^cre^;IRE1α^Δ/Δ^ (*n* = 6) mice were restimulated ex vivo with α-CD3/CD28 for 3 h, prior to addition of actinomycin D. Data represent two independent experiments pooled. Error bars show the mean ± s.e.m. *p* < 0.05 determined by linear regression analysis and ANOVA, n.d. not detected

To examine whether IRE1α serves to regulate stabilization of cytokine mRNAs within activated iNKT sublineages, we FACS sorted NKT1, 2 and 17 sublineages from the spleens of control and IRE1α-deficient and expanded them in vitro as previously described^34^. Following expansion, resting iNKT sublineages were restimulated with anti-CD3/CD28 and then treated with actinomycin D to inhibit mRNA transcription. Here, the rate of mRNA decay is expressed as the percentage mRNA remaining after the zero timepoint of actinomycin D addition. As shown in Fig. 4a, linear regression analysis of the percentage change in cytokine mRNAs present in control and IRE1α-deficient iNKT cells following addition of actinomycin D revealed a significant difference in the rate of cytokine mRNA decay within IRE1α-deficient NKT1 and NKT17, but not NKT2 sublineages. It is noteworthy that an elevated mRNA decay rate was also observed for the chemokine CCL3 within NKT1 cells, but not CCL5. Furthermore, we found no significant differences in the decay rates for several mRNAs commonly associated with “housekeeping” functions (e.g. *Actb*, Fig. 4a), suggesting that IRE1α regulates stability of mRNAs associated with iNKT sublineage effector function. These findings could be reproduced in expanded bulk splenic wild-type iNKT cells treated pharmacologically with the IRE1α inhibitors APY29 and B-109 (Fig. 4b, c). As observed for IRE1α-deficient iNKT cells, chemical inhibition of the kinase domain of IRE1α with APY29 resulted in significant mRNA decay rates for effector cytokines and chemokines relative to vehicle controls (Fig. 4b). Interestingly, chemical inhibition of the RNase domain of IRE1α with B-I09 does not affect the mRNA decay rates for effector cytokines and chemokines relative to vehicle controls (Fig. 4c). Importantly, decreased cytokine mRNA stability was also observed in IRE1α-deficient iNKT cells stimulated ex vivo relative to controls (Fig. 4e). However, the effects of APY29 and B-I09 on XBP1 splicing were maintained as described earlier^35,36^ (Fig. 4d). Thus, effector mRNAs within activated NKT1 and 17 sublineages are regulated by the kinase domain of IRE1α.

### An IRE1α-p38 MAPK pathway regulates cytokine mRNA stability

During ER stress, oligomerization of the luminal domain of IRE1α is associated with increased serine-threonine kinase activity within the cytosolic domain of the protein^9^. The kinase activity of IRE1α has been shown to be important in recruiting a signaling complex consisting of TRAF2 ^14^ or TRAF6 ^37^ and activation of the stress protein kinases c-Jun amino-terminal kinase (JNK) and p38 mitogen-activated protein kinase (MAPK), respectively^14,38,39^. Both JNK and p38 MAPK have been shown to regulate gene expression within the T-cell compartment by regulating cytokine mRNA stability^40,41^, suggesting that IRE1α might function within activated iNKT cells in a similar manner.

**Fig. 5:**
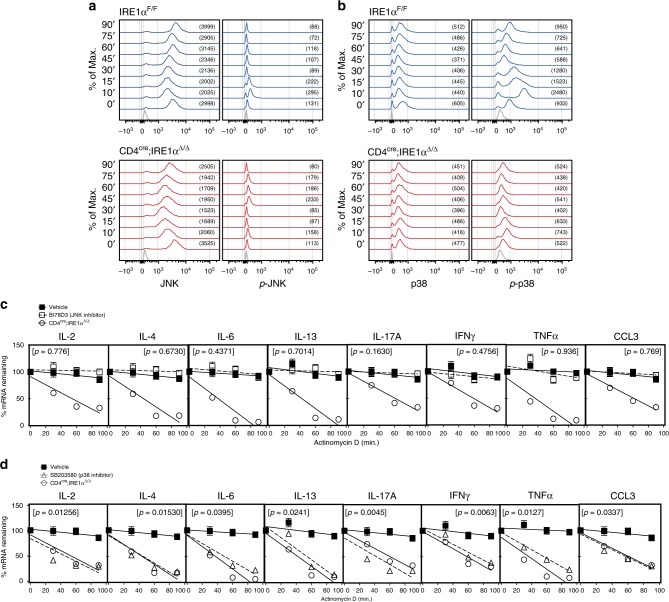
p38 MAPK regulates cytokine mRNA stability in iNKT cells. **a**, **b** Bulk splenic iNKT cells from IRE1α^F/F^ (*n* = 4) and CD4^cre^;IRE1α^Δ/Δ^ (*n* = 4) mice were expanded and restimulated with anti-CD3/CD28 from 0 to 90 min. Histograms represent intracellular expression of JNK, phospho-JNK, p38 and phospho-p38 in control and IRE1α-deficient iNKT cells following TCR restimulation, respectively. Isotype controls (gray) are shown at the bottom of the histogram overlays. Numbers in brackets represent MFI for JNK, p-JNK, p38 and p-p38 expression within FACS-gated iNKT cells. Histograms are representative of three independent experiments. **c**, **d** Purified bulk splenic wild-type iNKT cells were restimulated ex vivo with anti-CD3/CD28 for 3 h in the presence of the JNK inhibitor BI-78D3 (**c**) or the p38 inhibitor SB203580 (**d**), prior to addition of actinomycin D. Resting iNKT cells were then restimulated with anti-CD3/CD28 for 3 h and then treated with actinomycin D. mRNA decay rates were determined by linear regression analysis. Data represents three independent experiments pooled. Error bars show the mean ± s.e.m. *p* < 0.05 determined by linear regression analysis and ANOVA

We next examined whether IRE1α regulates activation of the JNK or p38 MAPK signaling pathways in TCR restimulated iNKT cells. For these experiments, control and IRE1α-deficient iNKT cells were FACS sorted from the spleens of IRE1α^F/F^ and CD4^cre^;IRE1α^Δ/Δ^ mice and expanded in vitro. Resting control and IRE1α-deficient iNKT cells were then restimulated with anti-CD3/CD28 and analyzed for intracellular expression of JNK, phosphorylated JNK (p-JNK), p38, and p-p38 by FACS. As shown in Fig. 5a, b, JNK and p38 MAPK protein kinases become phosphorylated in wild-type iNKT cells within the first 10 min following TCR restimulation. In contrast, IRE1α-deficient iNKT cells show a delayed phosphorylation kinetic for JNK (Fig. 5a), whereas phosphorylation of p38 is almost completely abolished when compared to control iNKT cells (Fig. 5b). To examine the functional relevance of JNK or p38 phosphorylation on cytokine mRNA stability in iNKT cells post TCR ligation, we restimulated wild-type iNKT cells in the presence of the JNK inhibitor BI-78D3^42^, or the p38 MAPK inhibitor SB203580^43^, prior to actinomycin D addition. Here, incubation of restimulated iNKT cells with the JNK inhibitor BI-78D3 had a negligible effect on cytokine mRNA stability when compared to vehicle controls (Fig. 5c) whereas p38 MAPK activity was found to be essential for maintaining stabilized mRNA transcripts in iNKT cells following TCR ligation (Fig. 5d). We therefore conclude that both JNK and p38 MAPK protein kinases are activated in iNKT cells post TCR-ligation; however, the regulation of p38 MAPK activity by IRE1α is needed to maintain cytokine mRNA stability following activation.

### IRE1α activity within iNKT cells regulates oxazolone colitis

**Fig. 6:**
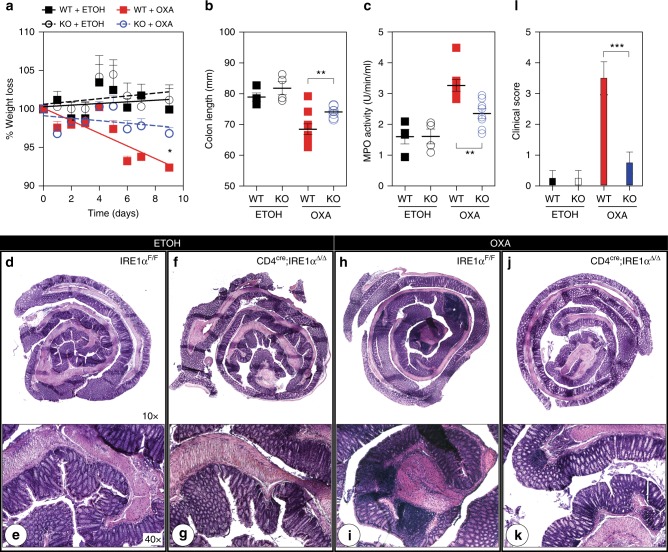
Deletion of IRE1α within iNKT cells protects mice from oxazolone colitis. IRE1α^F/F^ control (*n* = 10) and CD4^cre^;IRE1α^Δ/Δ^ (*n* = 10) mice were presensitized with either ethanol (ETOH) or 3% oxazolone (OXA) and then challenged intrarectally with either ethanol or 1% oxazolone 7 days after intrarectal challenge. Weight loss (**a**), colon length (**b**), MPO activity (**c**) and clinical score (**l**) were subsequently evaluated for each group after the intrarectal challenge. Data represent mean values obtained from two independent experiments. **d**, **f, h**, **j** Photomicrographs (×10) of hematoxylin and eosin (H&E)-stained sections of whole intestine removed from ethanol or oxazolone-treated control (**d**, **h**) and CD4^cre^;IRE1α^Δ/Δ^ (**f**, **j**) mice 5 days after intrarectal challenge. **e**, **g**, **i**, **k** Photomicrographs (×40) represent boxed distal regions of 10× H&E-stained sections representative for each group. Asterisk indicates areas of mucosal erosion. Error bars show the mean ± s.e.m. Mixed model analysis was performed to assess differences in weight loss and *p < 0.05, **p < 0.01, ***p < 0.001 determined by Mann–Whitney *U* test

The above data show that IRE1α is an important regulator of TCR-dependent cytokine production by iNKT cells. To evaluate whether these findings were relevant to the development of inflammation in a murine model of disease, we induced experimental colitis in wild-type IRE1α^F/F^ and CD4^cre^;IRE1α^Δ/Δ^ mice using oxazolone. Oxazolone is a haptenizing agent that induces Th2-driven mucosal inflammation in a manner dependent on CD1d and iNKT cells^44,45^. As shown in Fig. 6a, wild-type IRE1α^F/F^ mice that were rechallenged intrarectally with 1% oxazolone lost significantly more weight than did the oxazolone-treated CD4^cre^;IRE1α^Δ/Δ^ mice or ethanol-treated controls (Fig. 6a). Weight loss within rechallenged wild-type IRE1α^F/F^ mice was also associated with significantly reduced colon length (Fig. 6b), as well as increased myeloperoxidase activity within distal colon samples when compared to oxazolone-treated CD4^cre^;IRE1α^Δ/Δ^ littermate samples (Fig. 6c). In line with these findings, histopathology scores were elevated in oxazolone-treated wild-type IRE1α^F/F^ mice compared with CD4^cre^;IRE1α^Δ/Δ^ mice (Fig. 6l). Here, colons of oxazolone-treated wild-type IRE1α^F/F^ mice exhibited infiltration of inflammatory cells into the mucosa and lamina propria, as well as areas of mucosal erosion (Fig. 6h, i), that were not observed in oxazolone-treated CD4^cre^;IRE1α^Δ/Δ^ littermates or ethanol-treated control mice (Fig. 6d−k). These results therefore show that genetic deletion of IRE1α within iNKT cells protects mice from mucosal inflammation in the oxazolone colitis mouse model.

## Discussion

We propose that IRE1α, a UPR sensor, links key cellular processes involved in the regulation of cytokine production by activated iNKT cells. Results presented here show that TCR-mediated stimulation of the NKT1 and NKT17 sublineages results in autophosphorylation of IRE1α and subsequent splicing of XBP1, a UPR-specific transcription factor, that is involved in remodeling of the ER and preparing cells for enhanced cytokine secretion. Activation of the kinase domain of IRE1α promotes downstream activation of the p38 MAPK-signaling pathway which, in turn, regulates cytokine mRNA stability within the NKT1 and NKT17 sublineages. These findings provide a mechanistic link between the induction of ER stress in iNKT cells and the regulation of organ-specific inflammation in mice, as shown here using a murine model of intestinal inflammation.

Induction of ER chaperones and activation of the IRE1α-dependent UPR signaling arm in iNKT cells after TCR ligation is consistent with previous reports showing induction of UPR in TCR-stimulated T cells^31,46^. These studies did however not discriminate between CD4^+^ helper T cells and CD4^+^ iNKT cells when analyzing UPR activity within bulk CD4^+^ T-cell lineages. FlowSOM analysis of ERA1-transgenic mice performed in this study clearly shows highest expression of constitutively active IRE1α RNase within the iNKT cell lineage, particularly within peripheral iNKT cells, with little to no IRE1α RNase activity detected within the CD4^+^ or CD8^+^ T-cell compartments in steady-state mice. Furthermore, an analysis of TCR-stimulated TH-polarized CD4^+^ T cells in vitro showed no requirement for IRE1α in regulating cytokine mRNA expression or stability, arguing against a role for IRE1α-induced cytokine production in conventional CD4^+^ T-cell lineages. Although induction of an ER stress-response phenotype may be associated with priming of CD4^+^ and CD8^+^ T-cell lineages^21,22^, constitutive IRE1α RNase activity within peripheral NKT1 and NKT17 sublineages may be comparable with a cellular phenotype observed within specialized secretory cells. For example, terminal differentiation of mature B cells into plasma cells requires both IRE1α and XBP1 ^5,30^, and the upregulation of genes encoding secretory pathway components in plasma cells is coordinated by XBP1 ^12^. Similarly, an IRE1-XBP1 signaling axis is needed for development and differentiation of salivary gland acinar cells^47^, pancreatic beta cells^48^, and hepatocytes^49^. Although we found no requirement for IRE1α during iNKT sublineage specification and development, we propose that IRE1α-dependent signaling promotes NKT1 and NKT17 sublineage effector function in a manner analogous to the role of IRE1-XBP1 signaling during immunoglobulin production in plasma cells^5,30^. Similarly, TCR-dependent induction of ER-associated protein chaperones in NKT1 and NKT17 cells would facilitate increased cytokine protein folding and secretion both in vitro and in vivo. The unexpected finding that IRE1α regulates cytokine mRNA stability within restimulated NKT1 and NKT17 sublineages is, to our knowledge, the first dataset that describes a mechanistic link between the maintenance of preformed cytokine mRNAs within iNKT cells and their ability to rapidly produce cytokines following activation^3^. Although transcriptional regulation of *Il4* mRNA within iNKTs has been previously associated with chromatin accessibility of the *Il4* locus^2^, our data show that IRE1α-mediated regulation of several cytokine mRNAs is an essential mechanism controlling iNKT cell effector function. Importantly, incubation of iNKT cells with the kinase inhibitor APY29 is associated with decreased cytokine mRNA stability for in vitro expanded restimulated iNKT cells. However, decreased cytokine mRNA stability within iNKT cells was not affected in the presence of RNase domain inhibitor B-I09 (Fig. 4d). These findings are in agreement with a role for the kinase domain of IRE1α in the downstream activation of protein kinases such as JNK^14^. It remains to be determined how activation of IRE1α induces phosphorylation of p38 MAPK within iNKT cells; however, we have been able to formally exclude a role for either TRAF2 or TRAF6 in this signaling pathway (Supplementary Fig. 4A).

Our findings are in line with a previous report describing a role for MAPK kinase (MKK) 3, an upstream activator of the p38 MAPK pathway, in regulating cytokine production by iNKT cells^50^. MKK3, p38, and the downstream kinase MAPK-activated protein kinase (MAP-KAPK)-2 have been previously associated with the stabilization of inflammatory mRNAs^51^. In this context, a common characteristic of inflammatory mRNAs is the presence of AU-rich sequences in their 3′ untranslated regions. AU-rich elements (ARE) can act as potent mRNA destabilizing sequences as they can target mRNA for rapid deadenylation in vivo as well as promote 3′-5′ exonuclease decay^52^. It has been proposed that p38-mediated stabilization of inflammatory mRNAs is facilitated by a class of ARE-binding proteins (AREBP), such as TIA-1, which are functionally altered following activation of the p38 MAPK signaling pathway^53^. It remains to be determined whether IRE1α-p38-mediated stabilization of cytokine mRNAs within iNKT cells requires downstream modulation of AREBP function; however, it is interesting to note that TIA-1 has previously been shown to modulate IL-4 production within CD4^+^ T cells^22^.

Finally, the finding that CD4^cre^;IRE1α^Δ/Δ^ mice are protected from oxazolone colitis suggests that IRE1α-dependent signaling contributes to iNKT cell effector function in vivo as well. These results are significant as it suggests that IRE1α-mediated stabilization of cytokine mRNAs may be a general intracellular signaling cascade required for modulating iNKT cell effector function in vivo. Currently, a range of inflammatory, allergic and autoimmune diseases are believed to be modulated by cytokines secreted by iNKT cells in mouse and human. Although we were able to show that inhibition of IRE1α kinase function using APY29 inhibited cytokine mRNA stability in cultured iNKT cells, in vivo administration of APY29 to target iNKT cell effector function may have unwanted off-target side-effects such as inhibition of exocrine gland and liver function^47,49^. We therefore propose that a more detailed understanding of the mechanism by which IRE1α regulates cytokine production in iNKT cells may yield novel insights into signaling pathways that can be pharmacologically targeted to either inhibit or augment iNKT cell-driven disease.

## Methods

### Transgenic mice

Tg(CAG-XBP1*/Venus)^#Miur^ (ERAI-transgenic) mice have been previously described^23^. T-cell-specific IRE1α-deficient mice (CD4^cre^;IRE1α^Δ/Δ^) were generated by intercrossing mice carrying a LoxP flanked IRE1α allele^33^ with CD4-cre transgenic mice^34^. These transgenic mice were intercrossed with CXCR6^gfp^ mice for intravital imaging of iNKT cells as previously described^32^ All mice were housed and bred according to the guidelines of the Ghent University vivarium. All animal procedures were approved by the Institutional Animal Care and Ethics Committee.

### Flow cytometry and antibodies

Cells were analyzed on FACSCantoII (BD Biosciences) and FlowJo software (Tristar), and sorted on FACS Aria III (BD Biosciences). Antibodies used were CD122- PerCP-eFluor710 (TM-B1/46-1222-82/1:200), CD19-eFluor 450 (eBio1D3/48-0193-82/1:300), CD24-FITC (M1-69/11-0242-82/1:250), CD25-PE (PC61.5/12-0251-82/1:400), CD27-APC (LG.7F9/47-0271-82/1:150), CD3ε-V500 (500A2/560771/1:100/BD Biosciences), CD4-FITC/APC-eFluor780 (RM4-5/11-0042-82/17-00471-83/1:280), CD44-FITC/APC-eFluor780 (IM7/11-0441-85/25-0441-U100/1:275), CD62L-PE-Cy7 (MEL-14/60-0621-50U/1:600/TONBO), NK1.1-PE-Cy7 (PK136/25-5941-82/1:400), CD8α-V500 (53-6.7/560776/1:300/ BD Biosciences), βTCR-APC-eFluor780 (H57-597/47-5961-82/1:400), γδ ΤCR-PerCP-eFluor710 (eBioGL3/46-5711-82/1:250), IL2-PE-Cy7 (JES6-5H4/25-7021-80/0.25 μg), IL4-APC (11B11/17-7041-82/0.25 μg), IL6-FITC (MP5-20F3/561363/0.25 μg), IL13-PE-Cy7 (eBio13A//25-1733-80/0.25 μg), IFNγ-FITC (XMG1.2/11-7311-81/0.25 μg), TNFα-APC (MP6-XT22/17-7321-81/0.25 μg), T-bet-PE (eBio4B10/12-5825-82/0.25 μg), Gata-3-AlexaFluor488 (TWAJ/14-9966-82/0.25 μg), RORγt-PE (B2D/12-6981-80/0.25 μg), PLZF-AlexaFluor488 (Mags.21F7/53-9320-82/0.25 μg) (all other antibodies are from Invitrogen/eBioscience). p38α/β (A-12), JNK (D-2), β-Actin (from Santa Cruz Biotechnology, p-p38 MAPK-AlexaFluor 488 (28B10), p-JNK-AlexaFluor 647 (G9), IRE-1α (14C10/3294/1:1000), and EIF2α (9722/1ː1000) from Cell Signaling). α-GalCer-loaded CD1d tetramers (1:400) and MR1-5-OP-RU-APC (NIH/1:800). iNKT cells were stained at 4 °C using α-GalCer-loaded CD1d tetramers. MAIT cells were stained at room temperature using MR1–5-OP-RU tetramer^+^ provided by NIH tetramer core facility, USA. Stainings for intracellular proteins were performed using the FoxP3/Transcription Factor staining buffer set (eBioscience) for iNKT cell transcription factors or methanol for protein kinases according to the manufacturers’ recommendations. T lymphocyte subsets are defined as DN (CD3ε^−^CD4^−^CD8^−^); DP (CD3ε^−^CD4^+^CD8^+^); CD4^+^ T (CD4^+^CD8^−^CD1d-αGC^−^TCRβ^+^); CD8^+^ T (CD4^−^CD8^+^CD1d-αGC^−^TCRβ^+^); iNKT (TCRβ^+^CD1d-αGC^+^); NKT-Like (TCRβ^+^CD1d-αGC^−^NK1.1^+^), MAIT (MR1–5-OP-RU tetramer^+^ TCRβ^+^) and γδ^+^ T (TCRγδ^+^TCRβ^−^). TCRβ^+^CD1d-αGC^+^ NKT sublineages are defined as NKT1 (CD27^+/−^IL-2Rβ^+^), NKT2 (CD27^+^IL-2Rβ^−^), and NKT17 (CD27^−^IL-2Rβ^−^).

### FlowSOM analysis

FlowSOM analysis was performed as described previously^24^. Briefly, T-cell subsets were manually gated and the data for all the selected cells were aggregated per tissue. FlowSOM analysis used intensity measurements for NK1.1, CD4, α-GalCer-loaded CD1d tetramer, βTCR, γδ ΤCRand CD8α to group the cells into 100 nodes where node size indicates the mean percentage of cells assigned to the nodes. For analysis of *Venus*^FP^ expression, the median *Venus*^FP^ expression was computed for each node per sample. The final color value assigned to the node corresponds to the difference between the mean of the values obtained for ERAI-transgenic and wild-type mice, respectively.

### Cell suspension preparations

Single cell suspensions from the thymus were resuspended in phosphate-buffered saline (PBS) containing 1 mM ethylenediaminetetraacetic acid (EDTA) and 0.5% bovine serum albumin (BSA) while spleen and liver suspensions were layered over Ficoll or 33% Percoll gradients, respectively. Peripheral blood mononuclear cells (MNCs) were isolated by cardiac puncture in 20 U/ml PBS/heparin. iNKT thymocytes were enriched for using mouse CD8-Dynabeads (Invitrogen) and resuspended in 2 ml PBS containing 1 mM EDTA and 0.5% BSA.

### iNKT cell isolation, expansion, and restimulation

iNKT cells were FACS sorted from CD5-enriched MNCs isolated from the spleens or livers of pooled C57Bl/6, IRE1α^F/F^ or CD4^cre^;IRE1α^Δ/Δ^ mice as described earlier^54^. Briefly, α-GalCer/CD1d tetramer^+^ TCRβ^+^ iNKT cells present within the lymphocyte were gated after eliminating cell doublets, dead cells, CD19^+^ and γδT lymphocytes from the analysis by plotting FSC-H versus FCS-W, DAPI, CD19 (eFluor450), γδTCR (PerCp-Cy5.5) versus FSC-A, respectively (Supplementary Fig. 5). For ex vivo stimulation experiments, FACS-sorted iNKT cells were allowed to rest for 3 h after which 0.5 × 10^6^ iNKT cells were resuspended in complete RPMI 1640 medium (10% heat inactivated FCS, 100 U/ml penicillin/streptomycin, 2 mM glutamine, 0.1 mM nonessential amino acids, 5.5 × 10^2^ µM µ-ME, all from GIBCO) and stimulated with anti-CD3ε/CD28 (3 μg/ml, 5 μg/ml; eBioscience). For in vitro expansion experiments, sorted splenic bulk iNKT or NKT sublineages were resuspended in complete RPMI 1640 medium containing murine IL-2 (10 ng/ml), IL-12 (1 ng/ml), and soluble anti-CD28 (1 µg/ml; all from eBioscience) and stimulated with plate-bound anti-CD3ε (3 µg/ml) for 2 days. iNKT cells were then rested for 2 days and resuspended in complete medium containing murine IL-7 (2 U/ml; eBioscience) for 4 days. iNKT cells were then restimulated with plate-bound α-CD3ε (3 µg/ml) for a further 3 days and subsequently expanded for an additional 10 days in the presence of IL-7 and plate-bound anti-CD3ε, as described above. For restimulation experiments, 1 × 10^6^ iNKT cells were resuspended in complete RPMI 1640 medium (10% heat inactivated fetal calf serum (FCS), 100 U/ml penicillin/streptomycin, 2 mM glutamine, 0.1 mM nonessential amino acids, 5.5 × 10^2^ µM µ-ME, all from GIBCO) and stimulated with either PMA/Ionomycin (1 μM/200 ng/ml, Sigma-Aldrich), recombinant murine IL-12p70 (0.1 μg/ml, eBioscience), recombinant murine IFNβ (0.1 mg/ml, eBioscience), LPS (20 ng/ml; Sigma-Aldrich), Poly I:C (10 ng/ml; Sigma-Aldrich), 10 µg/ml murine CD1d (Sino Biological Inc.) loaded with 100 ng/ml α-GalCer or anti-CD3ε/CD28 (3 μg/ml, 5 μg/ml). For cytokine measurements, supernatants were collected after 72 h of stimulation and measured by ELISA.

### Immunoblot analysis and Phos-tag gels

Phosphorylation of IRE1α was monitored by Phos-tag SDS gels according to the manufacturer’s instructions (NARD Institute). Expanded bulk splenic iNKT cells (2 × 10^6^ cells) were stimulated with anti-CD3ε/CD28 (3 µg/ml, 5 µg/ml) for 0, 15, 30 and 60 min. Cells were spun for 10 min at 400 × *g at* 4 °C. After centrifugation, the medium was removed and washed with cold PBS. Cells were lysed for 15 min on ice in E1A buffer (1% NP40, 20 mM HEPES, pH 7.9, 250 mM NaCl, 1 mM EDTA, and protease and phosphatase inhibitors). Insoluble material was discarded by cold centrifugation, and a fixed amount of lysate was mixed with sample buffer before separation by sodium dodecyl sulfate–polyacrylamide gel electrophoresis (SDS-PAGE). Samples were separated using 6% SDS-PAGE containing 50 μM Phos-tag (NARD Institute) and 50 μM MnCl_2_. Gels were soaked in 1 mM EDTA before transfer for 10 min and transferred onto 0.45 μM polyvinyldifluoride membranes (Millipore). After transfer to a membrane, proteins were visualized by enhanced chemiluminescence kit (Thermo scientific).

### Polarization and restimulation of naïve CD4^+^ T cells

Naïve CD4^*+*^ T cells (CD4^+^TCRβ^+^CD1d-αGC^−^NK1.1^−^CD44^−^CD62L^hi^) were FACS sorted from the pooled spleens of IRE1α^F/F^ (*n* = 5) and CD4^cre^;IRE1α^Δ/Δ^ mice (*n* = 5), resuspended in complete RPMI 1640 media containing polarizing cytokines and plated at 1 × 10^5^ cells/well in 96-well plates coated with anti-CD3ε/α-CD28 (3 μg/ml, 5 μg/ml). Plated CD4^*+*^ T cells were differentiated under TH1-polarizing conditions using recombinant murine IL-12 (10 ng/ml, eBioscience), recombinant murine IL-2 (10 ng/ml, eBioscience), and anti-IL-4 (10 μg/ml, eBioscience); TH2-polarizing conditions using recombinant murine IL-4 (10 ng/ml, eBioscience), recombinant murine IL-2 (10 ng/ml) and anti-IFNγ (10 μg/ml, eBioscience); and TH17-polarizing conditions using recombinant murine IL-6 (20 ng/ml, eBioscience), recombinant murine IL-23 (10 ng/ml),^55^, recombinant murine IL-1β (10 ng/ml, PSF, VIB), recombinant human TGFβ1 (2 ng/ml, eBioscience), anti-IL-4 (10 μg/ml), anti-IFNγ (10 μg/ml) and anti-IL-2 (10 μg/ml, eBioscience), respectively. Cells were then removed from TCR stimuli after 3 days, resuspended at 1 × 10^6^/ml in fresh media and cultured in a new 96-well plate without TCR stimuli for 2 days. 1 × 10^6^ polarized T cells were then resuspended in complete RPMI 1640 medium and restimulated using anti-CD3ε/CD28 (3 μg/ml, 5 μg/ml; eBioscience).

### In vivo stimulation of iNKT cells

For serum cytokine production, 5 μg of α-GalCer in 200 ml PBS was injected intraperitoneally. Blood was subsequently collected at by retro-orbital puncture at 4 h postinjection and assayed for cytokine content by ELISA. For intracellular cytokine measurements, 2 μg of α-GalCer in 200 ml PBS was injected intraperitoneally and splenic iNKT cells were analyzed 2 h later by flow cytometry. For intravital imaging, 1 μg of α-GalCer in 20 μl PBS was injected intravenously. For in vivo labeling with BrdU (Sigma-Aldrich), the mice were injected i.p. with 1 mg BrdU (in PBS), followed by injection of 2 μg of α-GalCer intraperitoneally. At the same time, BrdU (1 mg/ml) was placed in the drinking water till the end of the experiment. Five days later, splenic iNKT cells were analyzed for BrdU incorporation using a BrdU flow kit (BD Biosciences) according to the manufacturer’s instructions.

### Intravital microscopy and image analysis

Multiphoton imaging of iNKT cells in the liver was performed as described before^56^. Briefly, to establish a baseline iNKT cell speed, 2 h of steady-state images were recorded prior to injection with α-GalCer. Imaging was continued for 2 h postinjection. Images were analyzed with Imaris (Bitplane) using ImarisTrack to quantify iNKT cell speeds over time. Cell velocities were plotted as a moving average of ten frames for each timepoint. Tracks of individual iNKT cells, normalized to their starting coordinates, were plotted with Matlab 9.1 (MathWorks). The square of the displacement distance over time was shown at steady-state and 1 h postinjection. Finally, a confinement index was calculated by multiplying cell track straightness by the square root of the track duration, both at steady-state and 1 h postinjection.

### Quantitative real-time RT-PCR

Total RNA was extracted from T lymphocytes analyzed in this study using the miRNeasy Mini kit (QIAGEN, Valencia, CA). cDNA was produced and amplified using the QuantiTect Whole Transcriptome Kit (QIAGEN, Valencia, CA). PCR products were amplified with the Fast Start SYBR Green Master mix (Roche, Basel, Switzerland) and detected with a LightCycler 480 System (Roche, Basel, Switzerland). Samples were normalized using qBasePlus (Biogazelle NV, Belgium) against at least three of the following housekeeping genes: *Rpl13a*, *Atp5b*, *Eif4b*, *Cyc1*, *B2m*, *Ubc*, *Actb*, *Gapdh*, *Sdha* or *Tubb4a*. Primer sequences for housekeeping, cytokine, ER chaperone and UPR signature genes are listed in Supplementary Table 1.

RNA stability assays iNKT and differentiated CD4^+^ T-cell subsets were stimulated with α-CD3ε/CD28 (3 µg/ml, 5 µg/ml) for 3 h after which 3 µg/ml of actinomycin D (Sigma-Aldrich) was added. RNA was subsequently isolated from actinomycin D-treated iNKT cells using the miRNeasy Mini kit (QIAGEN, Valencia, CA). Cytokine mRNAs were then normalized against at least three housekeeping genes expressed as the % mRNA remaining after the zero timepoint of actinomycin D addition. The slope of the line for % mRNA remaining between 0 and 90 min post-actinomycin D addition was determined by linear regression for each group or treatment condition. For inhibitor studies, anti-CD3ε/α-CD28-stimulated iNKT cells were incubated in the presence of 20 µM APY29 (4865, Tocris Bioscience), 20 µM B-109 (kindly provided by Andrew’s lab), 12.5 µM B1-78D3 (Sigma-Aldrich), 2.5 µM SB-203580 (Sigma-Aldrich) or equivalent vehicle controls prior to the addition of actinomycin D.

### Induction of oxazolone colitis

Eight-week-old mice were presensitized with 200 µl of a 3% (w/v) solution of oxazolone (4-ethoxymethylene-2-phenyl-2-oxazoline-5-one, Sigma-Aldrich) in 100% ethanol or 100% ethanol alone (vehicle) on the abdominal skin. Five days later mice were anesthetized with a mixture of ketalar, rompun and PBS and rechallenged intrarectally with 150 µl of either 1% (w/v) oxazolone in 50% ethanol or 50% ethanol alone (vehicle).

*Histology*: Mice were euthanized 10 days after induction of oxazolone colitis. The colons were subsequently removed and fixed with 4% formalin (Sigma-Aldrich). After paraffin embedding 7 µm sections were cut and stained with hematoxylin–eosin.

### Tissue myeloperoxidase activity

Samples of the mid-to-distal colon (adjacent to the samples used for histology) were rinsed with cold PBS, blotted dry, and immediately frozen in liquid nitrogen for storage at − 80 °C. Myeloperoxidase (MPO) activity was determined using the O-dianisidine method.

### Statistical analysis

Statistical testing was performed using the unpaired Student’s *t* test unless otherwise indicated. The Mann–Whitney *U* test was applied when data were demonstrated to not follow a Gaussian distribution, which is indicated in the respective figures legends. Linear regression analysis and ANOVA were performed for mRNA stability assays. Mixed model analysis was performed to assess differences in weight loss. All *p* values were two-tailed, and statistical significance was assumed at *p* < 0.05. For intravital imaging, linear mixed models for repeated measures were used except for the speed distribution, for which binomial logistic regression was performed.

## Supplementary information

Supplementary Information

Description of Additional Supplementary Files

Supplementary Movie 1

Supplementary Movie 2

## Data Availability

The data that support the findings in this study are available from the corresponding author upon request.
