# Supplementary material for: Stabilization of cytokine mRNAs in iNKT cells requires the serine-threonine kinase IRE1alpha
Source: Nat Commun. 2018 Dec 17;9:5340. doi: 10.1038/s41467-018-07758-x (PMC6297233; doi:10.1038/s41467-018-07758-x)
Supplement: Supplementary file 1 — Supplementary Information [file 41467_2018_7758_MOESM1_ESM.pdf]

SUPPLEMENTARY INFORMATION

Stabilization of cytokine mRNAs in iNKT cells requires the serine-threonine kinase IRE1alpha

Govindarajan et al.

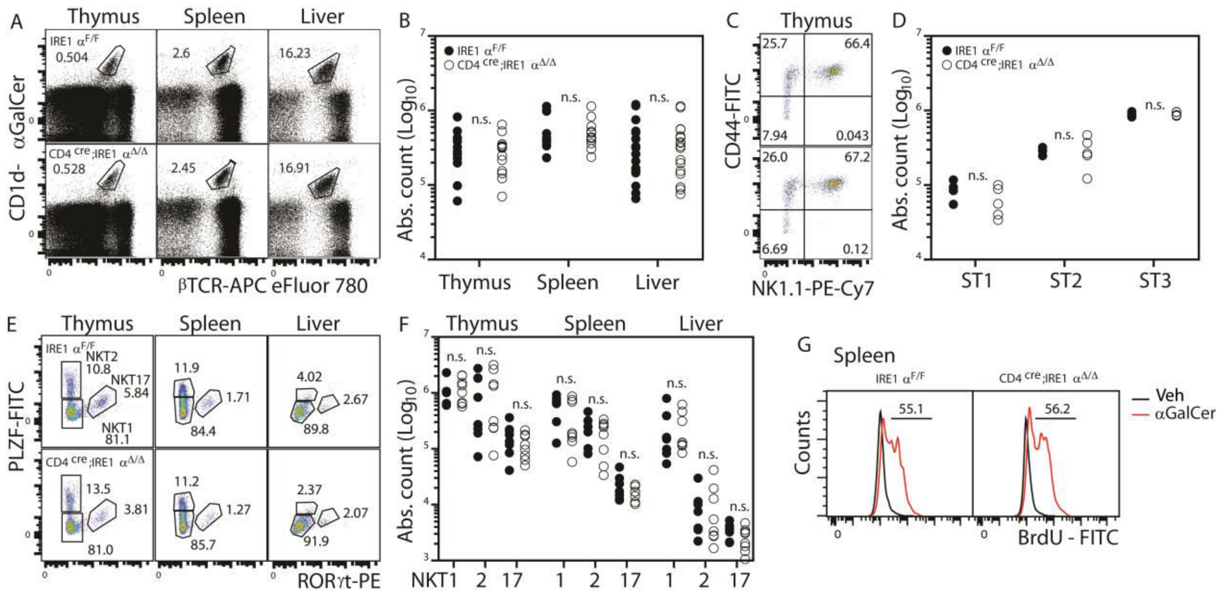

Supplementary Figure 1: Requirement for IRE1α during iNKT cell development. (**A**, **B**) Frequency and absolute cell iNKT cell counts within thymus, spleen and liver of control IRE1$\alpha^{F/F}$ (n = 13) and CD4^{cre};IRE1$\alpha^{\Delta/\Delta}$ (n = 12) mice, respectively. (**C**, **D**) Frequency and absolute cell counts of CD44^{-}NK1.1^{-} (Stage 1, ST1), CD44^{+}NK1.1^{-} (ST2) and CD44^{+}NK1.1^{+} (ST3) iNKT cells isolated from thymi of control (n = 6) and CD4^{cre};IRE1$\alpha^{\Delta/\Delta}$ (n = 5) mice, respectively. Flow cytometry plot and scatter dot blot represents two independent experiments pooled. (**E, F**) Frequency and absolute cell counts of NKT1 (PLZF$^{-/lo}$RORγt^{-}), NKT2 (PLZF$^{+/hi}$RORγt^{-}) and NKT17 (PLZF$^{-/lo}$RORγt^{+}) sublineages present in the thymus, spleen, and liver of control (n = 8) and CD4^{cre};IRE1$\alpha^{\Delta/\Delta}$ (n = 11) mice, respectively. Flow cytometry plot and scatter dot blot represents two independent experiments pooled. n.s., not significant. (**G**) Histogram represents marked increase in expansion and proliferation of iNKT cells both in control (n = 3) and CD4^{cre};IRE1$\alpha^{\Delta/\Delta}$ (n = 3) mice as measured by BrdU uptake 5 days after injection with α-GalCer (Red line) or Vehicle (Black line) respectively. Histogram represents two independent experiments. Error bars show the mean ± s.e.m. *$p < 0.05$ determined by Mann–Whitney U-test.

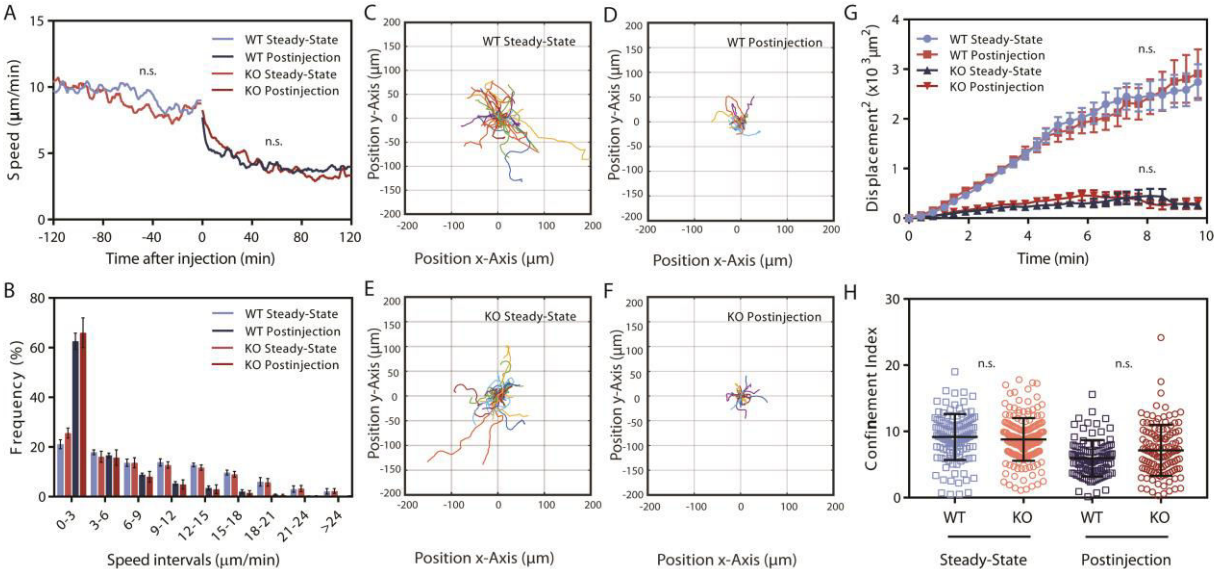

Supplementary Figure 2: Effect of IRE1α deficiency on iNKT cell motility. (**A**) Average iNKT cell speed over time within liver of control CXCR6$^{gfp/-}$IRE1α$^{F/F}$ (n=5) and CXCR6$^{gfp/-}$CD4^{cre};IRE1α$^{\Delta/\Delta}$ mice (n=3) at steady-state and after injection with α-GalCer. (**B**) Distribution of instantaneous iNKT cell speeds over different speed intervals for these mice. (**C-F**) Superimposed iNKT cell tracks of a representative mouse (**C;** CXCR6$^{gfp/-}$IRE1α$^{F/F}$ steady state mice, **D;** CXCR6$^{gfp/-}$IRE1α$^{F/F}$ post α-GalCer injected mice, **E;** CXCR6$^{gfp/-}$CD4^{cre};IRE1α$^{\Delta/\Delta}$ steady state mice, **F;** CXCR6$^{gfp/-}$CD4^{cre};IRE1α$^{\Delta/\Delta}$ post α-GalCer injected mice), normalized for the starting coordinates. (**G**) Displacement squared over time for cells tracks at steady-state and 1 hour postinjection (**H**) Confinement index at steady-state and 1 hour postinjection. Error bars show the mean ± s.e.m. A lack of statistical significance ($p > 0.05$) between WT and KO conditions is indicated as n.s (not significant).

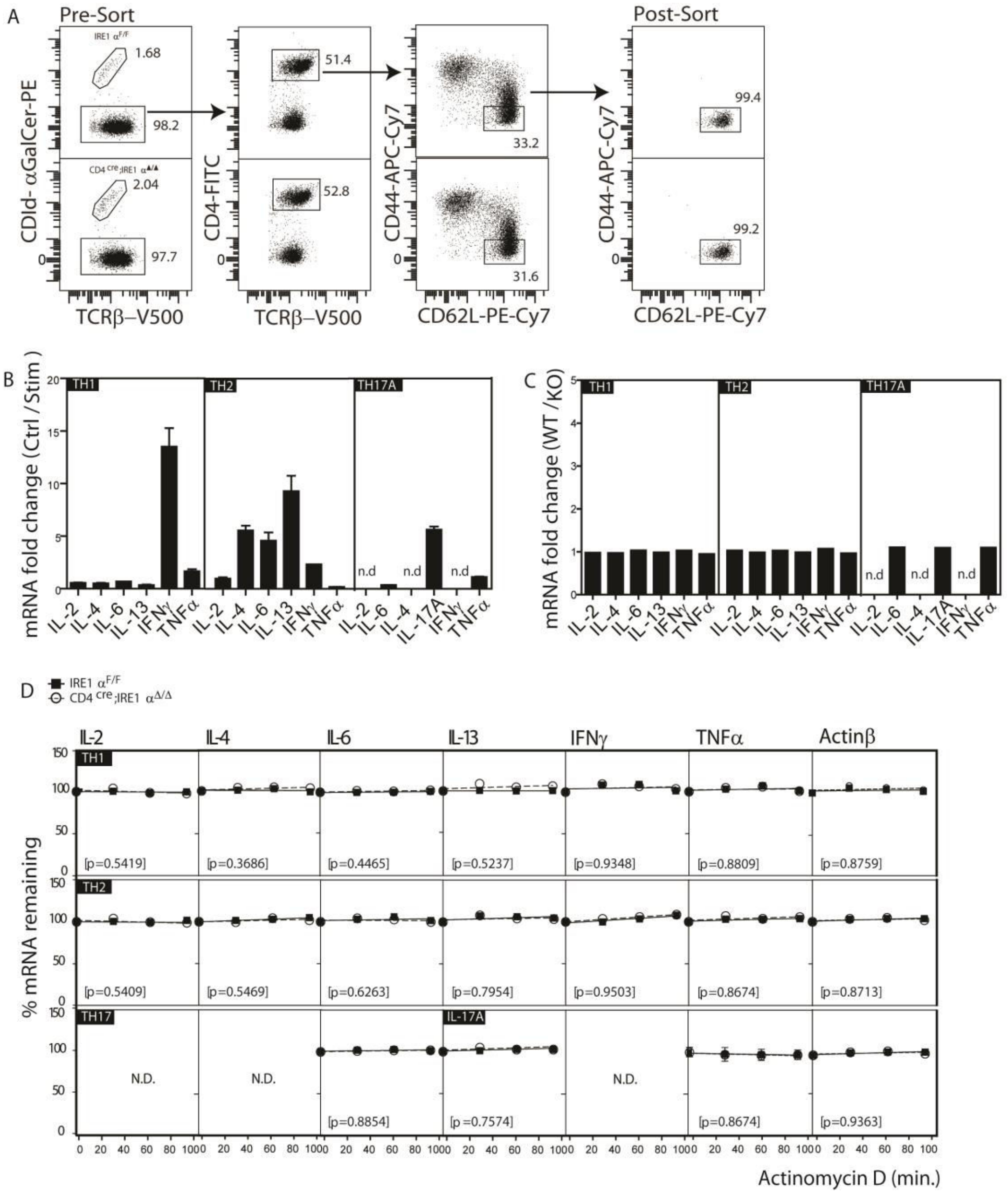

Supplementary Figure 3: Cytokine Production by conventional CD4^{+} T Cells is IRE1α-independent. (A-D) Naïve CD4^{+} T cells were FACS sorted from CD5-enriched MNCs isolated from pooled spleens of control (n = 6) and CD4^{cre};IRE1α$^{\Delta/\Delta}$ (n = 6) mice. Differentiated Th1, Th2 and Th17 CD4^{+} T cells in vitro, subsequently re-stimulated with anti-CD3/CD28 for 3 hours to analyze the cytokine gene expression and mRNA stability, respectively. (**A**) Percentage of naïve CD4^{+} T cells of pre-and post-sort within the TCRβ^{+}NKT^{-}CD4^{+}CD44^{-}CD62L^{hi} gate is shown in the far right of the plots. Flow cytometry plot represents 1 of 2 independent experiments. (**B-C**) qPCR analysis of fold-change in cytokine mRNAs produced by control or IRE1α-deficient TH-polarized CD4^{+} T cells following 3hrs of stimulation with anti-CD3/CD28. Bar chart represents three independent experiments pooled (**D**) Cytokine mRNA levels were then analyzed by qRT-PCR at 0, 15, 30 and 90 mins following addition of actinomycin D and normalized against the 0 time-point of actinomycin D addition. Data represents three independent experiments pooled. Error bars show the mean ± s.e.m. *$p < 0.05$ determined by linear regression analysis and ANOVA.

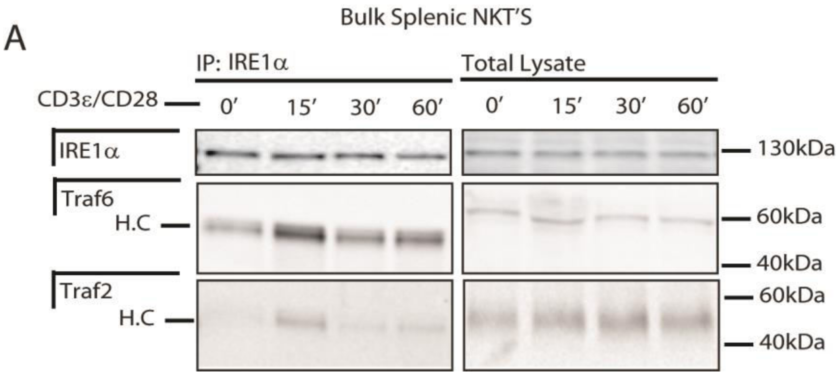

Supplementary Figure 4: Activation of p38 MAPK activity by kinase domain of IRE1α is independent of TRAF2 and TRAF6. The interactions between IRE1α and TRAF6 or TRAF2 were determined by immunoprecipitation and western blot analysis. TRAF6 protein in the lysates of TCR restimulated splenic expanded iNKT cells at indicated time points were immunoprecipitated with anti-IRE1α antibody; the TRAF6 and IRE1α were determined by western blotting with anti-TRAF6 Abs (midddle panel) and anti- IRE1α Abs (upper panel) respectively. The expression levels of IRE1α and TRAF6 in whole cell lysates were confirmed by western blotting with anti- IRE1α and anti-TRAF6 antibody respectively. Similarly the TRAF2 protein in the lysates of TCR restimulated splenic expanded iNKT cells at indicated time points were immunoprecipitated with anti-IRE1α antibody and subjected to western blot (lower panel) as mentioned above using anti- IRE1α and anti-TRAF2 antibody respectively; The data were from three independent experiments. H.C represents the reduced heavy chain of IRE1α antibody.

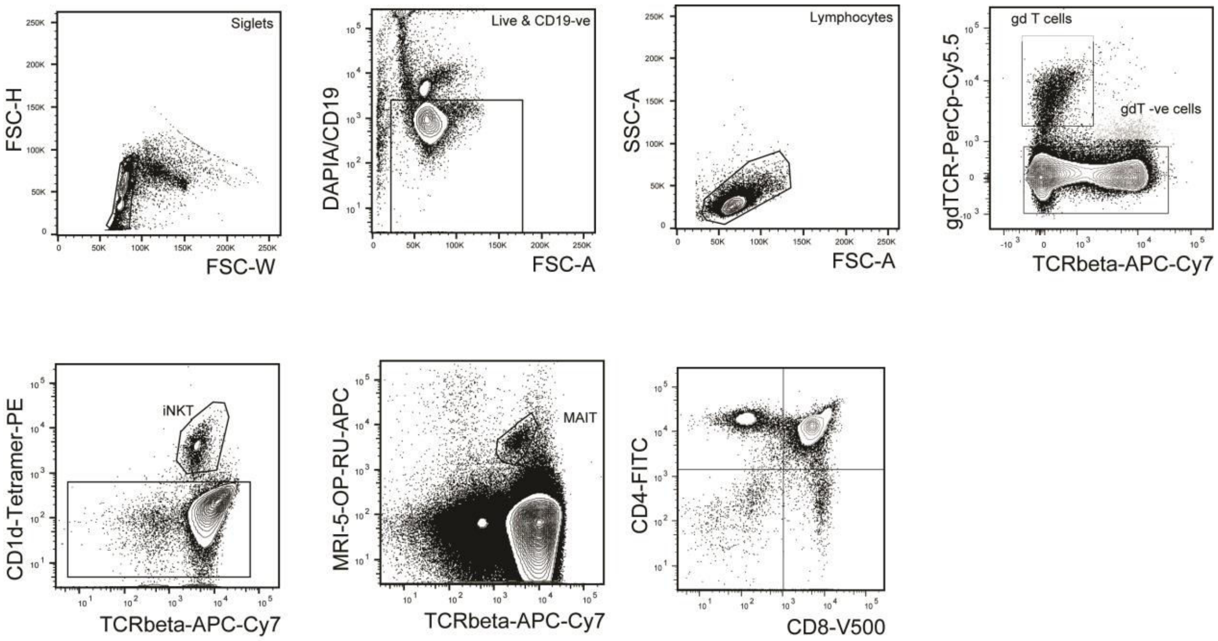

Supplementary Figure 5: Gating strategy for T lymphocytes: Flow cytometry plot represents the sequential gating strategy for analysing thymi T sublineage lymphocytes (singlets, Live/CD19- , lymphocytes, $\gamma\delta^+$ T (TCR$\gamma\delta^+$TCRβ^-), iNKT (TCRβ^+CD1d-αGC$^+$), MAIT (MR1–5-OP-RU tetramer$^+$ TCRβ^+), DN (TCRβ^-CD4$^-$CD8$^-$), DP (TCRβ^-CD4$^+$CD8$^+$), CD4$^+$ T (CD4$^+$CD8$^-$CD1d-αGC$^-$TCRβ^+), and CD8$^+$ T (CD4$^-$ CD8$^+$CD1d-αGC$^-$TCRβ^+).

Supplementary Table 1: Quantitative PCR primers

NM_009438	Rpl13A	GTGGTCGTACGCTGTGAAGGCATC	GGCCTCGGGAGGGGTTGGTATT
NM_016774	Atp5b	CCACCCGGATTCCGCCATGT	AGGATGAACCCCGGCGGGAG
NM_145625.3	Eif4al	CCCCCTCGCGCTGCTTTACG	GCGACGGGCTTGGAGACGTG
NM_025567.2	Cyc1	AACGGCGGCCGCACTAAAGG	CCTGCGGTGTCCGCAATGGA
NM_009735.3	B2m	ATGGCTCGCTCGGTGACCCT	TTCTCCGGTGGGTGGCGTGA
NM_019639	Ubc	ACAACTCCGTGAGAGAGACGATGC	GTAGTCTGACAGGGTGCGGCC
NM_007393	Actb	ACCCGCGAGCACAGCTTCTTTG	ACATGCCGGAGCCGTTGTCGAC
NM_008084	Gapdh	ACCCCAGCAAGGACACTGAGCAAG	TGGGGGTCTGGGATGGAAATTGTG
NM_023281.1	Sdha	CGGGCAGGCTCATCGGTGTT	TTCGCCCGTAGCCCCCAGTA
NM_009451.3	Tubb4a	CACTGCAAGCATCCTAGGCGGG	CCAGTGGGGTCGATGCCGTG
NM_008366.3	IL-2	GAATCCCAAACTCACCAGGA	TTATGTTTTCTCCCACCCCC
M25892.1	IL-4	GGTCTCAACCCCCAGCTAGT	GCCGATGATCTCTCTCAAGTGAT
M20572.1	IL-6	CTGCAAGAGACTTCCATCCAG	AGTGGTATAGACAGGTCTGTTGG
NM_008355.3	IL-13	TGAGCAACATCACACAAGACC	GGCCTTGCGGTTACAGAGG
NM_008355.3	IL-13	TGAGCAACATCACACAAGACC	GGCCTTGCGGTTACAGAGG
NM_010552.3	IL-17A	ACTACCTCAACCGTTCCACG	TTCCCTCCGCATTGACACAG
NM_008337.4	IFNgamma	ATGAACGCTACACACTGCATC	CCATCCTTTTGCCAGTTCCTC
BC137720.1	TNFalpha	CCCTCACACTCAGATCATCTTCT	GCTACGACGTGGGCTACAG
NM_013653.3	CCL5	GCAGTCGTGTTTGTCACTCG	CCGAGTGGGAGTAGGGGATT
NM_011337.2	CCL3	CCATATGGAGCTGACACCCC	GAGCAAAGGCTGCTGGTTTC
NM_013842	Xbp1u	AAACAGAGTAGCAGCGCACTGC	TCCTTCTGGGTAGACCTCTGGGAG
NM_013842	Xbp1s	TGCTGAGTCCGCAGCAGGTG	GCTGGCAGGCTCTGGGGAAG
NM_023913.2	Ern1	AACACACCGACCACCGTATC	AGGGTCCTGGGTAAGGTCTC
AF076681	Eif2ak3	GTTGGCACGGGAGAAGGTAA	TGAGTCACTGTTGTCTCCGC
NM_01081304.1	ATF6	GAACTTCGAGGCTGGGTTCA	TCCAGGGGAGGCGTAATACA
MMU89425	BIP	ATGACCCTACGGTGCAGCAGGAC	GGTTTGCCCACCTCCAATATCGGC
NM_007837	CHOP	ACCACACGGCGGGCTCTGAT	TCACATGCTTGGCGCTGGCG
BC033439	Pdia3	AAGGCTCTTGAACAGTTCCTGCAG	GGCCCTTCGTTGGACTCTGGGA
NM_013760	ERdj4	TGGGAGGAGGCTACTCGGCGTT	TTCTAATAACCTGTGCGCCCCGAC
NM_009787	Pdia4	GGCTTCCTTACCTCCAACCAA	TTCGCTACAGCAATGGGAGG
NM_011631	Hsp90b1	ACCGAAAAGGACTTGCGACT	AGCCTTCTCGGCTTTTACCC
