# Supplementary material for: Stabilization of cytokine mRNAs in iNKT cells requires the serine-threonine kinase IRE1alpha
Source: Nat Commun. 2018 Dec 17;9:5340. doi: 10.1038/s41467-018-07758-x (PMC6297233; doi:10.1038/s41467-018-07758-x)
Supplement: Supplementary file 2 — Description of Additional Supplementary Files [file 41467_2018_7758_MOESM2_ESM.pdf]

*Description of Additional Supplementary Files*

**Supplementary Movie 1: IRE1α-independent iNKT cell motility at steady-state**
Intravital time-lapse sequences of iNKT cells (green) in the liver at steady-state in a IRE1α WT (left) and a IRE1α KO (right) mouse. In both conditions iNKT cells are mostly motile. Blue strands correspond to collagen fibers that are present at the periphery of the liver, visualized by second harmonics. Bright blue auto fluorescent spots are retinoid-containing lipid droplets in hepatic stellate cells.

**Supplementary Movie 2: IRE1α-independent iNKT cell motility after αGalCer injection**
Intravital time-lapse sequences of iNKT cells (green) in the liver after injection of α-GalCer in a IRE1α WT (left) and a IRE1α KO (right) mouse. In both conditions, iNKT cells are mostly stationary. Blue strands correspond to collagen fibers that are present at the periphery of the liver, visualized by second harmonics. Bright blue auto fluorescent spots are retinoid-containing lipid droplets in hepatic stellate cells.
